# Reference intervals of Cyfra21-1 and CEA in healthy adult Han Chinese population

**DOI:** 10.1016/j.plabm.2024.e00409

**Published:** 2024-05-26

**Authors:** Lidan Xing, Shuai Zhao, Shichao Gao, Xiaoqian Shi, Yaomeng Huang, Puhuan Bian, Jingna Sun

**Affiliations:** Department of Clinical Laboratory, The First Hospital of Hebei Medical University, 89 Donggang Road, Yuhua District, Shijiazhuang, 050031, Hebei Province, China

**Keywords:** Reference interval, Cyfra21-1, CEA, Lung cancer, Healthy adults

## Abstract

**Objective:**

This study aimed to establish the reference intervals of Cyfra21-1 and CEA for the local screening populations using a chemiluminescence method.

**Methods:**

A total of 4845 healthy adults and 190 lung cancer patients were included from the First Hospital of Hebei Medical University. The levels of Cyfra21-1 and CEA were measured to establish the local reference intervals.

**Results:**

The upper limit reference intervals for Cyfra21-1 and CEA were determined as 3.19 ng/ml and 3.13 ng/ml, respectively. Notably, both Cyfra21-1 and CEA levels were found to be higher in males than in females. Additionally, both biomarkers showed an increasing trend with age.

In terms of diagnostic efficacy, the receiver operating characteristic (ROC) curve areas for Cyfra21-1, CEA, and their combination in lung cancer were 0.86, 0.73, and 0.91, respectively.

**Conclusion:**

Our study revealed that the reference intervals of Cyfra21-1 and CEA in the local population differed from the established reference intervals. Furthermore, both biomarkers exhibited gender-dependent variations and demonstrated a positive correlation with age. Combining the two biomarkers showed potential for improving the diagnosis rate of lung cancer.

## 1 Introduction

Cancer poses a significant and evolving challenge to both developed and developing countries worldwide. Among the various types of cancer, including lung cancer, breast cancer, gastric cancer, colorectal cancer, liver cancer, esophageal cancer, cervical cancer, ovarian cancer, bladder cancer, pancreatic cancer, and others, certain types are often diagnosed at an advanced stage due to their lack of evident early symptoms, as is the case with lung cancer. Particularly in recent times, the impact of the COVID-19 pandemic has brought increased attention to lung cancer. Non-small cell lung cancer (NSCLC) accounts for approximately 85 % of all lung cancer cases, while small cell lung cancer (SCLC) comprises the remaining 15 % [1]. As a leading cause of cancer-related mortality [2], lung cancer has been the subject of extensive research by scientists worldwide. Despite the identification and utilization of consistent diagnostic methods and biomarkers [3,4] for the detection and diagnosis of lung cancer, the 5-year survival rate for this disease remains dishearteningly low [5]. Consequently, there is an urgent need for regular and early population screening, particularly among high-risk individuals, utilizing relevant biomarkers.

Cyfra21-1, also referred to as cytokeratin fragment 19, is predominantly present in the alveolar epithelium. When altered, it can be released and enter the bloodstream, particularly in cases of squamous cell carcinoma of the lung and carcinoid tumors [6]. Interestingly, Cyfra21-1 represents a common tumor biomarker not only in lung cancer but also in gastrointestinal tumors and even severe alcoholic hepatitis [7]. Given their significance, researchers have increasingly focused on investigating the role of Cyfra21-1 in the diagnosis and prognosis of non-small cell lung cancer in recent years [6,8,9]. On the other hand, the carcinoembryonic antigen (CEA) family, consisting of 34 genes, initially known as biomarkers for colorectal cancer, has also been employed for lung cancer detection alongside other biomarkers such as Cyfra21-1. It is worth noting that clinical laboratory tests serve not only for disease diagnosis, management, and treatment but also for health screening purposes [10]. In this regard, Cyfra21-1 and CEA are no exceptions. Therefore, to enhance the accuracy of lung cancer screening in populations characterized by higher mortality rates, lower survival rates, and greater treatment challenges [11], it is imperative to establish more precise reference intervals for Cyfra21-1 and CEA.

However, it is important to acknowledge that the levels of Cyfra21-1 and CEA can be influenced by a multitude of factors, including ethnicity, geographical location, living customs, and the specific detection systems employed. Consequently, the reference intervals for Cyfra21-1 and CEA may vary across different regions. Given this variability, establishing appropriate and dependable reference intervals for the healthy adult population becomes an urgent imperative.

## 2 Materials and methods

### 2.1 Study population

This prospective study aimed to establish reference intervals for Cyfra21-1 and CEA using a cohort comprising 4397 healthy participants. From March 2022 to February 2023, a total of 2225 participants were assigned to the Cyfra21-1 group, while the remaining participants were allocated to the CEA group. Additionally, 448 healthy individuals and 190 patients with lung cancer were recruited to evaluate the diagnostic efficacy of lung cancer. All participants were admitted to the First Hospital of Hebei Medical University, located in China. The age of the participants ranged from 18 to 90 years, with a median age of 48 years. Prior to their involvement, this study received approval from the ethics committee at the First Hospital of Hebei Medical University, and all patients and their families provided informed consent. The collection of blood samples strictly adhered to relevant standards and protocols.

### 2.2 Inclusion and exclusion criteria

Patients included in the lung cancer group were diagnosed based on pathological examination and met specific exclusion criteria, which encompassed the absence of autoimmune diseases, acute and chronic infectious diseases, other significant organ diseases, or primary malignant tumors apart from lung cancer.

### 2.3 Samples collection

All blood samples were obtained from participants at the health examination center, following an overnight fast, between the hours of 8:00 a.m. and 9:30 a.m. Within 30–60 min after collection, the serum was separated from the blood by centrifugation at 400×*g* for 10 min. The subsequent analysis was performed within 4 h of sample collection. In cases where analysis could not be completed on the same day, the serum was carefully stored in a refrigerator at a temperature range of 2–8 °C, and the analysis was conducted within 48 h to ensure sample integrity.

### 2.4 Analysis of Cyfra21-1 and CEA testing

Cyfra21-1 levels in all samples were quantified using chemiluminescence on the MAGLUMI 4000 PLUS system (Shenzhen New Industries Biomedical Engineering Co., Ltd), with the appropriate calibration and quality control measures in place. Similarly, CEA levels in all samples were determined using chemiluminescence on the Wan 200+ system (Xiamen United Medical Instruments Co., Ltd), with the corresponding calibration and quality control measures implemented. It is important to note that all instruments and reagents utilized in this study were within their validity period.

### 2.5 Statistical analysis

**Fig. 1:**
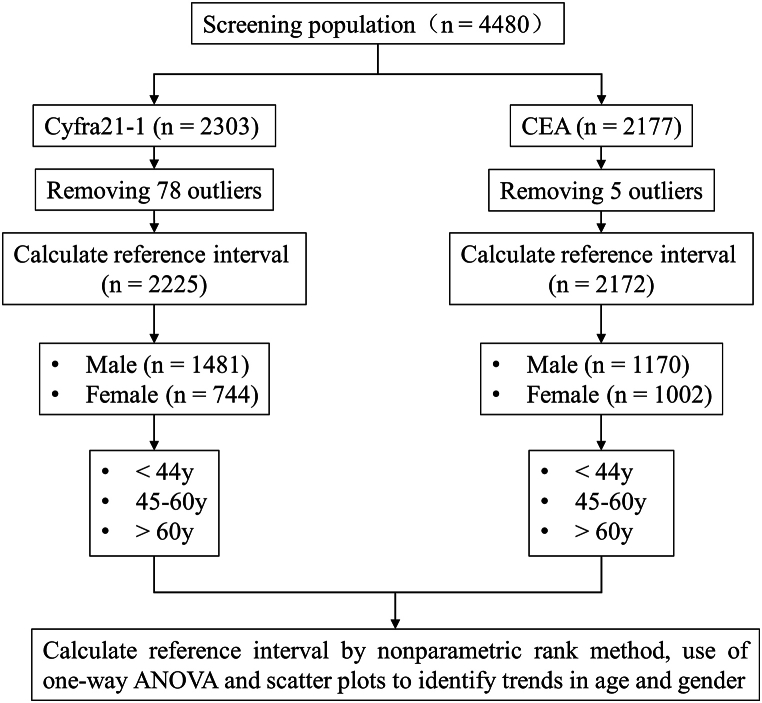
Establishment of reference intervals for Cyfra21-1 and CEA based on the data analysis protocol of CLSI guidance document CA28-A3.

Statistical analysis of the data was conducted using MedCalc software (version 17.0.4, Ostend, Belgium), IBM SPSS Statistics (version 25.0, SPSS Inc., Armonk, NY, USA), and GraphPad Prism (version 9.1.0, GraphPad Software; GraphPad, Bethesda, MD, USA), aligning with the guidelines outlined in CLSI CA28- A3. To analyze the data, a nonparametric percentile method was applied, specifically utilizing a 95 % right-sided approach. Outliers were identified through the Tukey test, while the Mann-Whitney test was employed for comparisons involving two groups, and the Kolmogorov-Smirnov test for comparisons involving three or more groups. Furthermore, 95 % confidence intervals (CI) for upper limits, as well as gender-related and age-related reference intervals were calculated, following the CLSI CA28- A3 guidelines (Fig. 1).

## 3 Results

### 3.1 Reference intervals of Cyfra21-1 for the apparently healthy adult population

A total of 2225 Cyfra21-1 tests were evaluated in this study, comprising 1481 male and 744 female participants, with an average Cyfra21-1 of (0.91 ± 1.07) ng/ml and (0.75 ± 0.92) ng/ml, respectively. The results of Cyfra21-1 exhibited an abnormal distribution, prompting us to establish reference intervals in accordance with the guidelines outlined in CLSI CA28-A3, which can be found in Table 1. The reference intervals for Cyfra21-1 among males and females were determined to be 3.29 ng/ml and 2.62 ng/ml, respectively. Detailed reference intervals for different age groups and genders can be found in Table 2. Specifically, for males, the reference intervals in three age groups were identified as 3.08 ng/ml, 3.36 ng/ml, and 3.71 ng/ml, while for females, they were determined to be 2.30 ng/ml, 2.85 ng/ml, and 2.42 ng/ml.

**Table 1:** Reference intervals of Cyfra21-1 and CEA for the healthy adult population.

Gender	Cyfra21-1 (ng/ml)				CEA (ng/ml)		
	n	Median	Upper limit		n	Median	Upper limit
Male	1481	0.45	3.29		1170	0.82	3.52
			90 % CI: 3.19–3.48				90 % CI: 3.27–3.75
Female	744	0.30	2.62		1002	0.29	2.52
			90 % CI: 2.43–2.85				90 % CI: 2.27–2.75
Total	2225	0.40	3.19		2172	0.57	3.13
			90 % CI: 3.03–3.29				90 % CI: 3.02–3.27

CI: confidence interval.

**Table 2:** Reference intervals of Cyfra21-1 for the different ages and gender.

Age (year)	Male				Female		
	n	Median	Upper limit		n	Median	Upper limit
≤44	559	0.28	3.08		270	0.15	2.30
			90 % CI:2.55–3.31				90 % CI:1.95–2.80
45–59	670	0.50	3.36		327	0.34	2.85
			90 % CI:3.16–3.91				90 % CI:2.58–3.45
≥60	252	0.82	3.71		147	0.54	2.42
			90 % CI:3.29–4.16				90 % CI:2.01–3.15

CI: confidence interval. The unit of Cyfra21-1 is ng/mL.

**Fig. 2:**
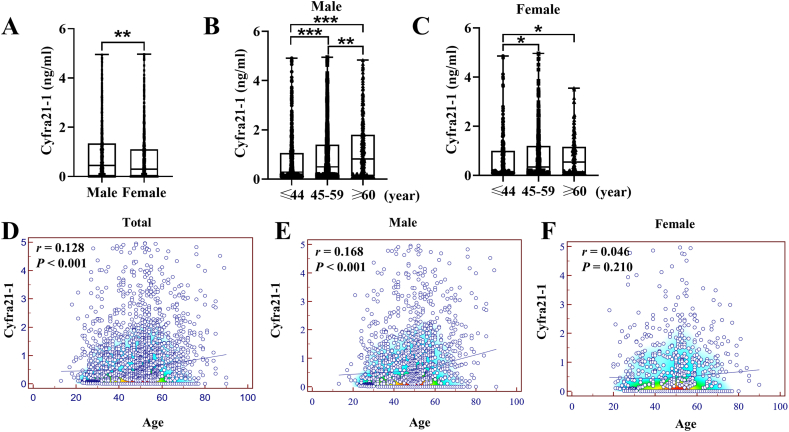
Distribution of Cyfra21-1 in different ages and gender. (A) Box plot of Cyfra21-1 in the male and female groups. (B) Box plot of Cyfra21-1 in different ages of the male group. (C) Box plot of Cyfra21-1 in different ages of the female group. (D) Scatter plots and Spearman's correlation between Cyfra21-1 and age. (E) Scatter plots and Spearman's correlation in different ages of the male group. (F) Scatter plots and Spearman's correlation in different ages of the female group. *P < 0.05, **P < 0.01, ***P < 0.001.

The results depicted in Fig. 2A demonstrate a significant disparity in Cyfra21-1 levels between males and females (P < 0.01), with males exhibiting considerably higher levels. Additionally, the analysis revealed a substantial elevation in Cyfra21-1 levels as age increased, as depicted in Fig. 2B and C (P < 0.05). Furthermore, a noteworthy positive correlation between Cyfra21-1 levels and age was observed, as depicted in Fig. 2D (*r* = 0.128, P < 0.001); however, this correlation was found exclusively among males, as indicated in Fig. 2E (*r* = 0.168, P < 0.001), while no significant correlation was detected among females, as depicted in Fig. 2F (*r* = 0.046, P = 0.210).

### 3.2 Reference intervals of CEA for the apparently healthy adult population

A total of 2172 CEA tests were evaluated in our comprehensive study, encompassing 1170 male and 1002 female participants, with an average CEA of (1.14 ± 1.19) ng/ml and (0.65 ± 0.91) ng/ml, respectively. The observed distribution of CEA results demonstrated an abnormal pattern, necessitating the establishment of reference intervals, which are outlined in Table 1. Specifically, the reference intervals for CEA among males and females were determined to be 3.52 ng/ml and 2.52 ng/ml, respectively. In Table 3, comprehensive reference intervals for CEA corresponding to different age groups and genders are provided. Among males, the reference intervals were established as 2.68 ng/ml, 3.63 ng/ml, and 4.38 ng/ml for the three age groups, while among females, they were identified as 1.55 ng/ml, 2.51 ng/ml, and 3.47 ng/ml for the equivalent age groups.

**Table 3:** Reference intervals of CEA for the different ages and gender.

Age (year)	Male				Female		
	n	Median	Upper limit		n	Median	Upper limit
≤44	509	0.57	2.68		503	0.02	1.55
			90%CI:2.44–2.87				90%CI:1.38–1.82
45–59	334	0.83	3.63		294	0.35	2.51
			90%CI:3.15–4.24				90%CI:2.17–2.82
≥60	327	1.39	4.38		205	0.99	3.47
			90%CI:3.95–5.02				90%CI:3.20–4.93

CI: confidence interval. The unit of CEA is ng/mL.

**Fig. 3:**
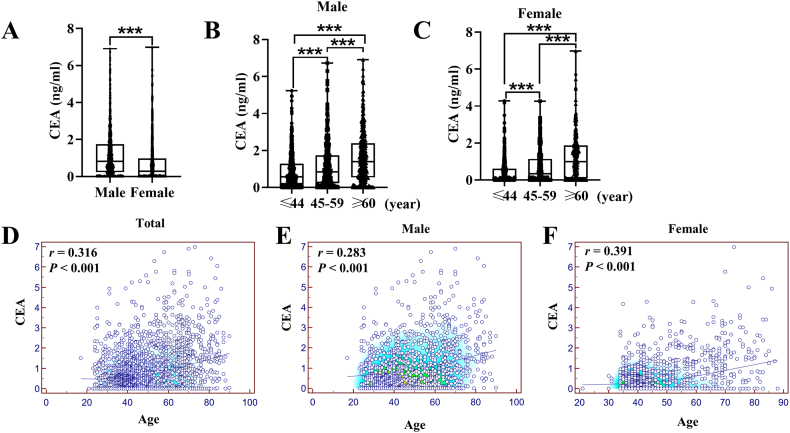
Distribution of CEA in different ages and gender. (A) Box plot of CEA in the male and female groups. (B) Box plot of CEA in different ages of the male group. (C) Box plot of CEA in different ages of the female group. (D) Scatter plots and Spearman's correlation between Cyfra21-1 and age. (E) Scatter plots and Spearman's correlation in different ages of the male group. (F) Scatter plots and Spearman's correlation in different ages of the female group. ***P < 0.001.

The findings presented in Fig. 3A provide robust evidence of a noteworthy disparity in CEA levels between males and females (P < 0.001), with significantly higher levels observed among males. Furthermore, a meaningful association between CEA levels and age was established, as depicted in Fig. 3B and C, with a significant elevation observed as age progressed (P < 0.001). Additionally, a substantial positive correlation between CEA levels and age was identified, as illustrated in Fig. 3D (*r* = 0.316, P < 0.001). Importantly, this correlation was observed in both males, as depicted in Fig. 3E (*r* = 0.283, P < 0.001), and females, as illustrated in Fig. 3F (*r* = 0.391, P < 0.001).

### 3.3 Diagnostic efficacy of Cyfra21-1 and CEA for lung cancer

**Fig. 4:**
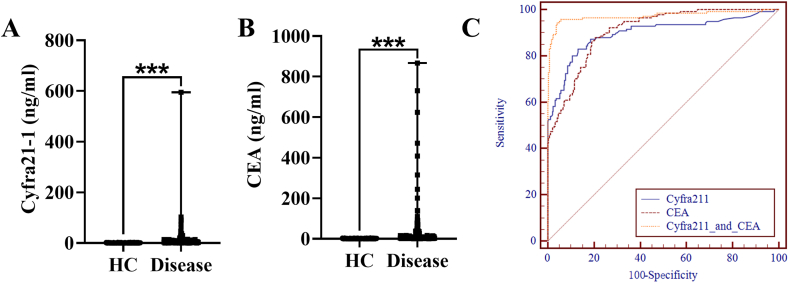
Diagnostic efficacy of Cyfra21-1 and CEA for lung cancer. (A) Levels of Cyfra21-1 in the health control (HC) and lung cancer (Disease) group. (B) Levels of CEA in the HC and Disease group. (C) The ROC curves illustrated the sensitivity and specificity of Cyfra21-1 and CEA in diagnosing lung cancer. The corresponding AUC values are listed in Table 5.

In order to assess the diagnostic efficacy of Cyfra21-1 and CEA in detecting lung cancer, a study involving 190 lung cancer patients (referred to as the Disease group) and 448 apparently healthy adults (serving as the Healthy Control group) was conducted. Median of Cyfra21-1 and CEA in different types of cancer were showed in Table 4. No statistically significant differences were observed between the Healthy Control and Disease groups in terms of age (P > 0.05) or gender (χ^2 = 0.247, P > 0.05). As illustrated in Fig. 4, the levels of Cyfra21-1 and CEA were found to be significantly elevated in the lung cancer group compared to the control group (Fig. 4A and B, P < 0.001). The area under the receiver operating characteristic (ROC) curve (AUC) values for Cyfra21-1, CEA, and the combined detection were determined to be 0.86, 0.73, and 0.91, respectively (Fig. 4C and Table 5). Importantly, the combined AUC was significantly higher compared to the individual markers of Cyfra21-1 and CEA alone (Table 5). The combined detection exhibited a sensitivity of 82.11 % and a specificity of 95.54 %. The identified cut-off values for Cyfra21-1 and CEA were 2.81 ng/ml and 2.44 ng/ml, respectively, which could serve as reliable diagnostic markers for lung cancer.

**Table 4:** Median of Cyfra21-1 and CEA in different types of cancer.

	LUAD (n = 93)	LUSC (n = 35)	NSCLC (n = 13)	*P*
Cyfra21-1	3.13 (2.29–5.095)	19.06 (12.02–38.30) *	0.09 (0.08–1.025) *^#^	<0.01
CEA	11.26 (4.125–40.375)	2.45 (2.22–2.72) *	1.64 (1.01–1.945)^#^	<0.01

NOTE: LUAD: Lung Adenocarcinoma; LUSC: Lung Squamous cell carcinoma; SCLC: small cell lung cancer.

*P < 0.05, vs. LUAD, ^*#*^*P* < 0.05, vs. LUSC.

**Table 5:** The ROC analysis of Cyfra21-1 and CEA in the diagnosis of lung cancer.

Biomarker	AUC	Sensitivity	Specificity	Cut-off value	95 % CI		P value
Cyfra21-1	0.86	76.84	97.32	2.81	0.813	0.893	<0.01
CEA	0.73	61.05	75.89	2.44	0.672	0.773	<0.01
Cyfra211 + CEA	0.91*^,#^	82.11	95.54	–	0.869	0.936	<0.01

*P < 0.05, Cyfra211 + CEA vs. Cyfra21-1 only; ^#^*P* < 0.05, Cyfra211 + CEA vs. CEA only.

## 4 Discussion

Despite numerous attempts to address lung cancer, which has emerged as a leading global cause of mortality [[12], [13], [14]], early diagnosis remains inadequate. Furthermore, lung cancer susceptibility is influenced by various factors beyond smoking, including occupational exposure, age, air pollution, and genetic factors [15,16]. Consequently, regular and timely screening is paramount in reducing the incidence of lung cancer and ensuring population health. To mitigate the risk of missed diagnoses and delayed treatments, we established reference intervals for Cyfra21-1 and CEA based on CLSI guidelines, employing percentiles and their corresponding confidence intervals. By determining the median and upper reference limits for lung cancer in our investigation, we aim to equip clinicians with biomarker values that facilitate prompt and accurate assessments of individuals.

Cyfra21-1 has emerged as a valuable biomarker for non-small cell lung cancer since its initial exploration. Elevated levels of Cyfra21-1 have been associated with a diminished 5-year survival rate for this type of cancer [17]. In our study, we observed that the values of Cyfra21-1 did not follow a normal distribution, prompting us to utilize a nonparametric method (specifically, a right-sided Tukey test with a 95 % confidence level) to analyze the reference intervals in accordance with CLSI EP28-A3 guidelines. Surprisingly, we found that the reference interval for Cyfra21-1 differed from the provided reference interval. Specifically, our study determined a Cyfra21-1 value of 3.19 ng/ml, which is lower than the given reference interval of 3.30 ng/ml. This discrepancy suggests that adhering to the provided reference interval may result in overlooking potential individuals at risk. Additionally, our analysis revealed that males exhibited higher levels of Cyfra21-1 compared to females. This discrepancy may be attributed to factors such as hormonal influences and metabolic variations, warranting further investigation. Furthermore, we observed that Cyfra21-1 levels increased with age and displayed a positive correlation, consistent with findings reported by Dubin et al. [15], who identified age as a risk factor for lung cancer. However, when the data was analyzed separately by gender, this trend was not observed, implying that age may impact Cyfra21-1 values among males to a greater extent than females. Consequently, establishing a localized reference interval for Cyfra21-1 is essential in our study population.

Despite its initial identification as a biomarker for colon cancer in 1965, CEA has increasingly been reported in association with other cancer types [17,18]. In our study, we also observed a non-normal distribution of CEA values, prompting us to employ the same analytical method to establish reference intervals specific to our study population. Surprisingly, we discovered that the reference interval for CEA (3.13 ng/ml) was considerably lower than the given reference interval (5.0 ng/ml). This discrepancy not only exhibited a significant difference in distribution between genders but also demonstrated a positive correlation with age, similar to the findings for Cyfra21-1. Both gender and age appear to influence the biomarkers' expression in some manner, highlighting the importance of establishing region-specific reference intervals for Cyfra21-1 and CEA in lung cancer screening.

The diagnostic efficiency of Cyfra21-1 and CEA in predicting lung cancer was evaluated by constructing receiver operating characteristic (ROC) curves. Our findings indicated that the combined use of Cyfra21-1 and CEA yielded a larger area under the curve (AUC) compared to their individual usage. Furthermore, the threshold values associated with Cyfra21-1 and CEA were lower than the established clinical reference value, suggesting that the combined approach significantly enhances the diagnostic efficiency of lung cancer. These results imply that utilizing both biomarkers in combination could improve the diagnostic accuracy for lung cancer, particularly when applied alongside appropriate local reference intervals for the target populations.

It is important to acknowledge the limitations of our study. Firstly, while our findings present promising results, the inclusion of supplementary biomarkers alongside clinical features of lung cancer may further enhance the diagnostic accuracy of the disease. Secondly, although the diagnostic efficacy of the two biomarkers investigated in our study may not be exceptionally robust, they do play a significant role in providing valuable insights for early population-based screening initiatives.

## 5 Conclusion

In our study, we successfully determined new reference intervals for Cyfra21-1 and CEA, revealing notable gender disparities and a positive correlation tendency with age. Moreover, our findings highlighted the potential of combining these two biomarkers to enhance the diagnostic efficiency of lung cancer.

## Journalism ethics considerations

Ethical issues (Including plagiarism, informed consent, misconduct, data fabrication and/or falsification, double publication and/or submission, redundancy, etc.) have been completely observed by the authors.

## CRediT authorship contribution statement

**Lidan Xing:** Writing – review & editing, Writing – original draft. **Shuai Zhao:** Validation, Supervision. **Shichao Gao:** Formal analysis. **Xiaoqian Shi:** Data curation. **Yaomeng Huang:** Methodology. **Puhuan Bian:** Data curation. **Jingna Sun:** Project administration, Funding acquisition.

## Declaration of competing interest

The authors declared that there was no conflict of interest.

## Data Availability

Data will be made available on request.
